# Whole genome sequencing and 6-year follow-up of a mother and daughter with frontometaphyseal dysplasia associated with keratitis, xerosis, poikiloderma, and acro-osteolysis: A case report

**DOI:** 10.1097/MD.0000000000011283

**Published:** 2018-07-13

**Authors:** Heng Xie, Li Xue, Wei Hua, Bangsheng Jia, Liang Zhang, Li Li

**Affiliations:** a Department of Dermatology; b Department of Radiology, West China Hospital; c State Key Laboratory of Oral Diseases & National Clinical Research Center for Oral Diseases & Department of Implant Dentistry, West China Hospital of Stomatology, Sichuan University, Chengdu, Sichuan, China.

**Keywords:** acro-osteolysis, congenital glaucoma, frontometaphyseal dysplasia, keratitis, poikiloderma, whole genome sequencing, xerosis

## Abstract

**Rationale:**

Frontometaphyseal dysplasia (FMD) is a dominant X-linked rare disease caused by mutations of *FLNA*. The distinctive features of FMD include skeletal dysplasia, facial dysmorphism, extremities anomalies, deafness, cleft palate and eye anterior segment anomalies, yet none of the complications, such as acro-osteolysis, keratitis, xerosis or poikiloderma, have been reported in FMD.

**Patient concerns:**

A 29-year-old mother and her 7-year-old daughter, both presented with congenital glaucoma, craniofacial dysmorphism, xerosis and poikiloderma, were admitted to our hospital in 2011. Additionally, the mother also suffered from acro-osteolysis, keratitis, camptodactyly of hands and metastatic cutaneous squamous cell carcinoma (SCC) which turned out to be fatal 5 years later. In 2017, keratitis and acro-osteolysis were noticed in the daughter as well. Radiography showed bowed long bones with thickening cortex, and distal phalangeal osteolysis.

**Diagnoses:**

Whole genome sequencing (WGS) was conducted in 2016, resulting in 71491 single-nucleotide polymorphisms and 7616 indels shared by patients while the father was taken as control. A *FLNA* variant was classified likely pathogenic, supporting the diagnoses of FMD. In addition, though our patients’ symptoms were highly consistent with xeroderma pigmentosum variant, a mild subtype of xeroderma pigmentosum (XP) with merely accumulated UV-induced lesions like xerosis and poikiloderma limited to sun-exposure sites, higher risks of cutaneous neoplasms and absence of classical XP features, WGS didn’t find supportive genetical evidence, but 2 *HERC2* variants were assigned highest suspicion in both XP and SCC by bioinformatical analyses.

**Interventions:**

Anti-inflammatory treatment, sunscreens and moisturizers were administered.

**Outcomes:**

The daughter's cutaneous lesions developed slowly during the 6-year follow-up, but the keratitis seriously weakened her sight.

**Lessons:**

To our knowledge, it's the first report of cases carrying FMD, keratitis, xerosis, poikiloderma and acro-osteolysis simultaneously, and 3 likely pathogenic variants were identified. Whole genome/exon sequencing is recommended as a common test for patients with rare phenotypes.

## 1 Introduction

Frontometaphyseal dysplasia (FMD; MIM305620) is a dominant X-linked rare disease belonging to otopalatodigital spectrum disorders (OPDSDs), all associated with *FLNA* mutations.^[1,2]^ With overlapping symptoms, OPDSDs are mainly featured by skeletal dysplasia (bowed long bones, scoliosis, etc.), facial dysmorphism (ocular hypertelorism, micrognathia, broad nasal bridge, etc.), extremities anomalies (distal phalangeal hypoplasia, long digits, absent halluces, etc.), deafness and cleft palate.^[1,2]^ Eye anterior segment anomalies including proptosis, glaucoma and sclerocornea are frequently observed.^[1]^ However, in FMD, deafness, cleft palate and scoliosis is less common and progressive contractures of hands could be found.^[1,2]^

Herein, we report a mother and daughter of Han Chinese within a 6-year follow-up, who presented with a complex of FMD, acro-osteolysis, keratitis, xerosis, and poikiloderma. By investigation via whole genome sequencing (WGS), genetical analyses and literature review, 3 likely pathogenetic variants were identified and the possibility of a new entity was proposed.

## 2 Case report

### 2.1 Case 1

**Figure 1:**
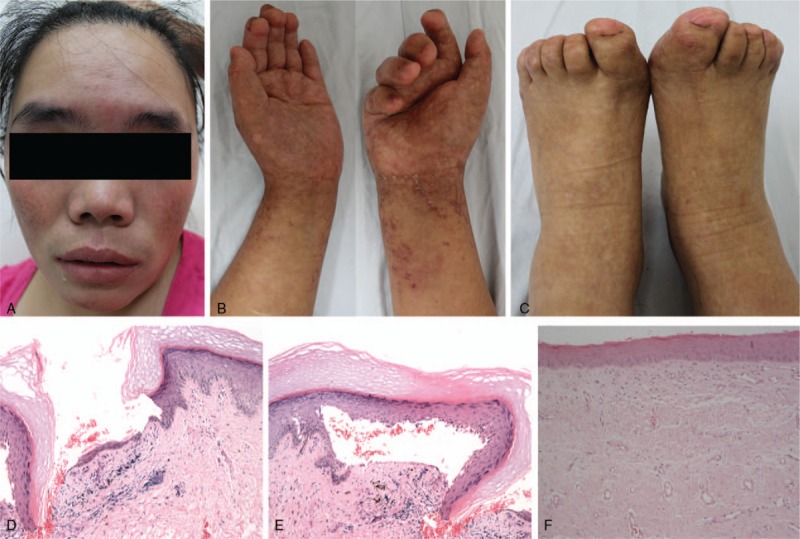
Telangiectasia, hyperpigmentation, keratitis, ocular hypertelorism, proptosis, broad nasal bridge, and nasal tip and full cheeks could be observed on the face (A). Xerosis, poikiloderma (telangiectasia, hyperpigmentation, hypopigmentation, atrophy) and camptodactyly existed on extremities (B, C) and hands were affected more severely (B). Skin biopsy (hematoxylin-eosin staining, 100×) from the mother's right hand showed: epidermal hyperkeratosis, local epidermal loss (D), mild acanthosis, focal liquefactive degeneration of basal layer, fissures beneath epidermis (E), telangiectasia and perivascular infiltration of dense lymphocytes and pigmentophages in superficial dermis (F).

The mother, aged 29 at the first visit in 2011, was blind soon after birth because of congenital glaucoma. Then poikiloderma gradually developed since her childhood: distal limbs’ skin became dry and telangiectasia, hyperpigmentation, hypopigmentation, and scales were observed; telangiectasia and hyperpigmentation were also found on bilateral cheeks (Fig. 1A and C). In this period, the grandmother died (none other maternal relatives could be found and none paternal kinsfolk exhibited positive phenotypes), but details could not be retrieved due to the long-term interval and blindness, which also led to incomplete memories on the occurrence of keratitis and shortened distal phalanges happening without paresthesias in her puberty (Fig. 1A). Camptodactyly of hands was noticed in 20s. The cutaneous lesions slowly developed during adolescence and adulthood. Linear atrophic lesions showed up on limbs (Fig. 1B and C). Skin biopsy at the first visit suggested chronic inflammation throughout epidermis and dermis (Fig. 1D–F). Three years later, the mother underwent an amputation of right lower limb because of metastatic cutaneous squamous cell carcinoma (SCC) originated from a recurrent ulceration at the right heel, and ultimately died of SCC's relapse in 2016. WGS and its analysis assigned the highest suspicion to *FLNA*, indicating the diagnosis of FMD.

### 2.2 Case 2

**Figure 2:**
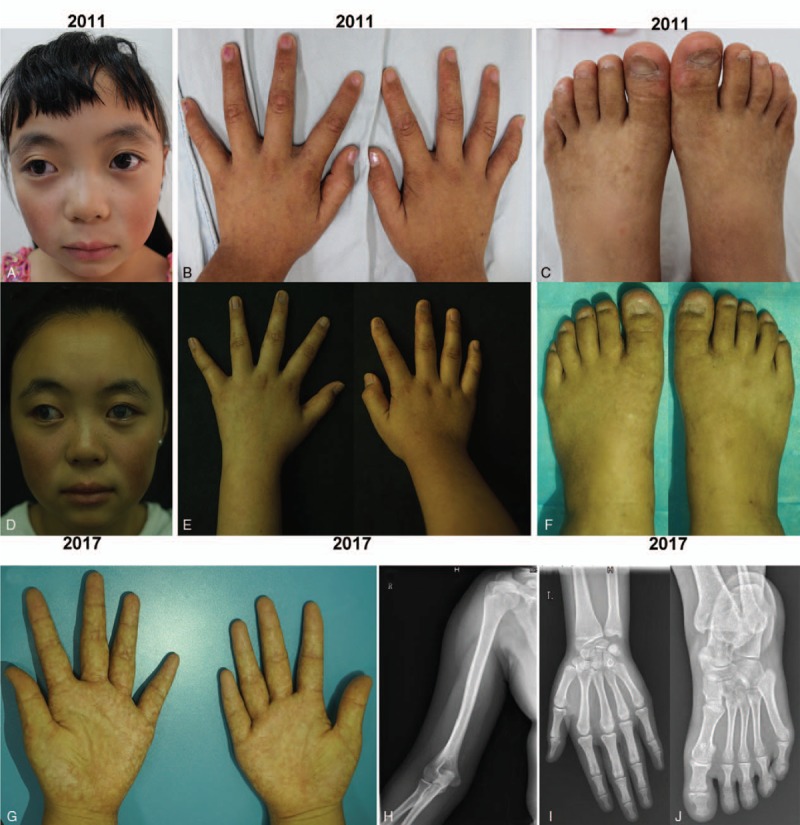
Facial dysmorphism, xerosis, and poikiloderma (A–C) were similar with her mother (Fig. 1A–C) when she was 7 in 2011. In addition, the daughter's toenails were malformed slightly (C) while shapes all the fingers and toes were roughly normal (B and C). At the age of 13 in 2017, though cutaneous lesions developed slowly (D–G), the patient's eyes were affected by emerging keratitis (D) while distal phalanges got thinner mildly (E–G) and nail abnormities, wrinkles on nails (E and F) and smaller toenails (F), were more marked. X-ray exanimations showed bowed humerus with thickened cortex (H–J) and distal phalangeal osteolysis.

The daughter, aged 7 in 2011, was also diagnosed of congenital glaucoma after birth. Though vision was preserved after timely surgery, the daughter still suffered from severe myopia, ocular hypertension, and photophobia. The clinical features of her skin were milder but consistent with her mother's (Fig. 2A–C). Some toenails were malformed but digits were roughly normal (Fig. 2B and C). Both of their craniofacies showed ocular hypertelorism, proptosis, broad nasal bridge, and nasal tip and full cheeks (Figs. 1 and 2). Their sunburn reactions, blood routine, immunologic test, hepatic and renal functions and radiography, dentition, palate, development and intelligence were within the normal range. In 2017, the daughter, at the age of 13, exhibited deteriorating poikiloderma, emerging keratitis, acro-osteolysis (no Raynaud's phenomenon) and loss of nails (Fig. 2D–G). X-ray showed the bowed long bones with thickening cortex, and distal phalangeal osteolysis (Fig. 2H–J). WGS in this girl also suggested the diagnosis of FMD. Patient-education of UV protection was performed and close follow-up was recommended. The poikiloderma did not obviously develop after utilizing anti-inflammatory treatment (compound glycyrrhizin), sunscreens and moisturizers.

## 3 Genetic analyses

Blood samples of the mother and daughter were obtained and the next-generation WGS was performed in 2016 by BGI Co., Ltd (Shenzhen, China), while the father's was taken as control. wANNOVAR, a nonprogramming bioinformatical tool, was utilized in annotation, prioritization, prediction and genotype–phenotype network of variants shared by the mother and daughter.^[3,4]^ Multiple in silico algorithms were adopted in prediction, including 5 popular predictors (SIFT, PolyPhen-2, LRT, MutationTaster, and Mutation Assessor) and a meta-score (metaSVM) integrating 11 common predictors.^[3,4]^ Phenotype key words (skin abnormities, extremities, glaucoma) were input to optimize genotype–phenotype networking. The candidate variants were validated by Sanger sequencing and their pathogenicity was classified by published guidelines.^[5]^ Ethical approval was waived approval was waived here and a patient consent was given.

**Figure 3:**
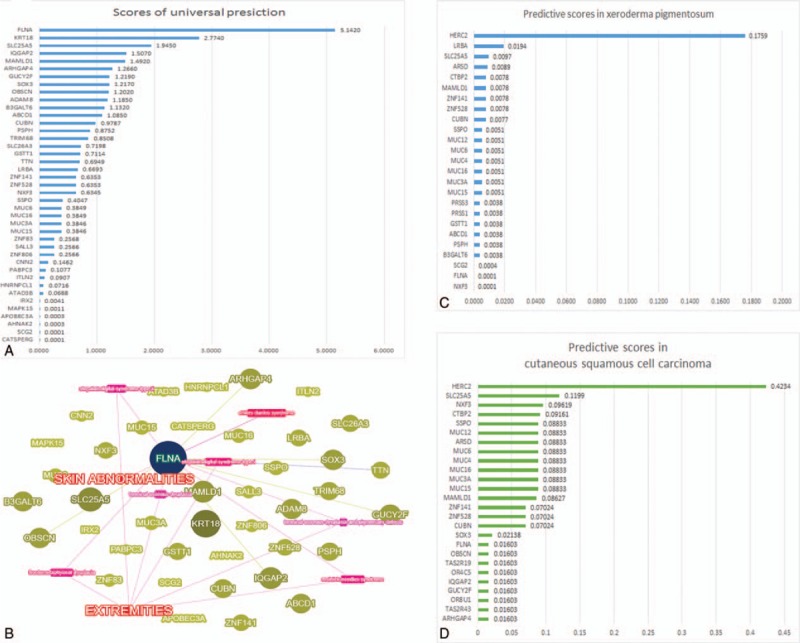
*FLNA* was granted highest suspicion in the prediction taking all phenotypes as a unity (A). It was also classified as a seed gene in the genotype–phenotype network (B): deep blue spot: seed gene; light yellow spot: predicted gene; pink rectangle: specific disease; red words: key words of phenotypes. *HERC2* got highest predictive scores of wANNOVAR in both predictions for candidate genes in xeroderma pigmentosum (C) and cutaneous squamous cell cancer (D).

In total, 71491 single-nucleotide polymorphisms (SNPs) and 7616 indels shared by patients were identified. Bioinformatical predictions and genotype–phenotype network upon the whole dataset and symptoms indicated *FLNA* as the causative gene (Fig. 3A and B). Based on literature review, clinical manifestations (facial dysmorphism, hands contractures, congenital glaucoma, the absence of deafness, cleft palate, scoliosis and short stature) and radiography (bowed long bones, thickened cortex), the diagnosis of FMD was established.^[1,2]^ Two *FLNA* mutations were identified, one of which, a missense variant (p.Val528Met/c.1582G>A) in exon 11, also detected in the father's X chromosome (the control), was considered benign. The other variant (c.4474+59G>A), on the other hand, lying at the board between exon 25 and intron 25, was classified likely pathogenic (Table 1).

**Table 1:**
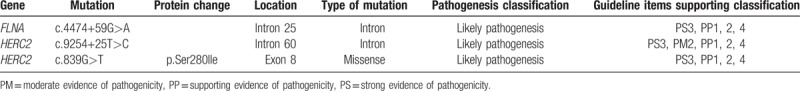
List of genes classified as pathogenic or likely pathogenic.

However, FMD could not explain symptoms like photophobia, keratitis, the parchment-like appearance (xerosis and poikiloderma), chronic cutaneous inflammation, and subsequent SCC, which, in fact, were highly consistent with xeroderma pigmentosum (XP) variant type (XPV; MIM278780). We identified 19 nonsilent SNPs across all XP-related genes, 18 of which, according to guidelines, were classified benign for their allele frequencies were over 5% while the left *POLH* variant (p.Met647Leu/c.1939A>T) located in the last exon of *POLH* (exon 11), was excluded for it also existing in the father.^[6–8]^ Then we checked the documented variants across all the xeroderma pigmentosum-Cockayne syndrome-trichothiodystrophy spectrum and found none positive results.^[8]^ Intriguingly, with the predictions of wANNOVAR, *HERC2* was granted the highest scores in both cutaneous XP and SCC (Fig. 3C and D). We ruled out 20 *HERC2* variants shared by the father, identified 2 likely pathogenic mutations (Table 1); however, little is known about the 2 variants.

## 4 Discussion

FMD has the lowest detection rate of *FLNA* mutation (57%), which is almost 100% in other OPDSDs, rendering establishing a diagnosis sometimes difficult, especially in females who usually present markedly attenuated phenotypes.^[1,2]^ According to our cases, we deduce this phenomenon may be resulted from widely used exon sequencing in previous studies leading to less detections on intron variants, which, located at certain sites, could also be disease causing.^[1,2,5,9]^

Xeroderma pigmentosum (XP) is an autosomal recessive dermatosis characterized by sun sensitivity, sunlight-induced ocular abnormalities, and high risk of cutaneous neoplasms. Most XP patients have mutations in *XPA* through *XPG*, and *XPV* (also named *POLH*), mainly involved in nucleotide excision repair of ultraviolet (UV) irradiation.^[6–8]^ Classical XP phenotypes include acute sunburn, persistent erythema on minimal sun exposure, noticeable freckles, photophobia, keratitis, skin lesions on lids, and even nervous abnormities, while patients carrying *POLH* variants, slightly affected in general, might only have accumulated UV-induced lesions like xerosis and poikiloderma limited to sun-exposure sites. These mild patients usually are not diagnosed timely and own a higher malignancy predisposition, especially to melanoma, SCC and basal cell carcinoma.^[6–8]^ Our patients’ symptoms showed high consistence with XPV and the effective anti-inflammatory treatment and sun-protection measures were also supportive evidence, but the heterogeneous *POLH* c.1939A>T mutation (already documented in documented in melanoma, nonsmall cell lung cancer, SCC and XPV) and the mutation's existence on the father do not support the diagnosis.^[10–12]^

Mutations of *HERC2*, affecting stabilization and chromatin retention of XP complementation group A (XPA), might also be involved in cutaneous SCC.^[13,14]^ XPA is controlled by circadian clock-mediated transcriptional regulation, while the ubiquitination mediated by homologous to the E6-AP carboxyl terminus domain and RCC1-like domain-containing protein 2 (HERC2), which leads to XPA's degradation, could also contributes to the circadian oscillation.^[14]^ A recent study, in addition, reported variants at the *OCA2/HERC2* locus have strong association between time to first cutaneous SCC post-transplant.^[13]^ Hence, though the diagnosis of XPV could not get genetical support, our patients’ *HERC2* variants may be helpful in unveiling the potential pathogenesis of both XP and SCC.

Acro-osteolysis is a representative characteristic of Hadju–Cheney syndrome (HCS; MIM102500), an autosomal dominant disease caused by mutations in the last exon of *NOTCH2* leading to a truncated protein.^[15,16]^ With features of progressive focal bone destruction and craniofacial anomalies, it often exhibits similar phenotypes with OPDSDs, thus acro-osteolysis, generalized osteoporosis, cystonephrosis, and genetic test are required in differing HCS from OPDSDs.^[17–20]^ Our cases’ *NOTCH2* mutations were checked. Excluding 10 variants also carried by the father, neither of the 2 left mutations lied in the last exon of *NOTCH2*. In addition, none of other characteristics apart from acro-osteolysis and facial dysmorphism were observed, suggesting a remote possibility of HCS.

None similar cases could be found after literature review. Generally, this could be just a coincidence of coexistence of multiple diseases or a new entity. Considering the high consistence between the mother and daughter in symptoms and results of genetic analyses, we are prone to the latter likelihood.

In summary, to our knowledge, it is the first report of cases presenting FMD, keratitis, xerosis, poikiloderma and acro-osteolysis simultaneously, and 3 likely pathogenic variants were identified. Whole genome/exon sequencing is recommended as a common test for patients with rare phenotypes. Even though it is hard to find out decisive conclusions from the small number of patients, accumulated reports could gradually enrich the dataset so that the identification of causative mutations and subsequent targeting treatment could be expected.

## Author contributions

**Conceptualization:** Heng Xie, Wei Hua, Li Li.

**Data curation:** Heng Xie, Li Xue, Bangsheng Jia, Liang Zhang, Li Li.

**Formal analysis:** Heng Xie, Liang Zhang.

**Investigation:** Heng Xie, Li Xue, Bangsheng Jia, Liang Zhang.

**Methodology:** Heng Xie, Wei Hua, Liang Zhang, Li Li.

**Project administration:** Li Li.

**Resources:** Li Xue, Wei Hua, Bangsheng Jia, Liang Zhang.

**Software:** Heng Xie, Bangsheng Jia.

**Supervision:** Wei Hua, Li Li.

**Validation:** Wei Hua, Liang Zhang, Li Li.

**Visualization:** Heng Xie.

**Writing – original draft:** Heng Xie.

**Writing – review & editing:** Wei Hua, Li Li.
